# Family group conferencing in youth care: characteristics of the decision making model, implementation and effectiveness of the Family Group (FG) plans

**DOI:** 10.1186/1471-2458-14-154

**Published:** 2014-02-11

**Authors:** Jessica J Asscher, Sharon Dijkstra, Geert Jan JM Stams, Maja Deković, Hanneke E Creemers

**Affiliations:** 1 Forensic Child and Youth Care Sciences, University of Amsterdam, Nieuwe Prinsengracht 130, 1018 VZ Amsterdam, The Netherlands; 2 Child and Adolescent Studies, Utrecht University, Utrecht, The Netherlands

**Keywords:** Effectiveness, Randomized controlled trial, Family Group Conferencing, Child safety, Supervision order, Perceived control, Social network, Professional help

## Abstract

**Background:**

The model of Family group-conferencing (FG-c) for decision making in child welfare has rapidly spread over the world during the past decades. Its popularity is likely to be caused by its philosophy, emphasizing participation and autonomy of families, rather than based on positive research outcomes. Conclusive evidence regarding the (cost) effectiveness of FG-c is not yet available. The aim of this protocol is to describe the design of a study to evaluate the (cost) effectiveness of FG-c as compared to Treatment as Usual.

**Method/Design:**

The effectiveness of FG-c will be examined by means of a Randomized Controlled Trial. A multi-informant approach will be used to assess child safety as the primary outcome, and commitment of the social network, perceived control/ empowerment; family functioning and use of professional care as secondary outcomes. Implementation of FG-c, characteristics of family manager and family will be examined as moderators of effectiveness.

**Discussion:**

Studying the effectiveness of Fg-c is crucial now the method is being implemented all over the world as a decision making model in child and youth care. Policy makers should be informed whether the ideals of participation in society and the right for self-determination indeed result in more effective care plans, and the money spent on FG-c is warranted.

**Trial registration:**

Dutch Trial Register number NTR4320. The design of this study is approved by the independent Ethical Committee of the Faculty of Social and Behavioral Sciences of The University of Amsterdam (approval number: 2013-POWL-3308). This study is financially supported by a grant from ZonMw, The Netherlands Organization for Health Research and Development, grant number: 70-72900-98-13158.

## Background

The model of Family group-conferencing (FG-c) for decision making in child welfare has rapidly spread over the world during the past decades [1]. Also in the Netherlands, Family Group conferences are widely used as a decision making procedure in youth care [2]. Research has indicated, however, that its popularity is likely to be caused by its philosophy (emphasizing participation and autonomy for families), rather than based on research outcomes showing positive results for FG-c [3,13]. The question is whether FG-c’s in youth care yield what they are developed for: increased child’s safety (i.e., decrease in (risk for) abuse/neglect, less supervision orders or shorter supervision order, less or shorter out of home placement), involvement of the broader social network; perceived control over the problems in the families, empowerment of parents; improvement of family functioning, and less use of professional care.

Despite the broad implementation of FG-cs globally and despite many research efforts into FG-c, Frost et al. (2012) concluded that it is not possible to draw conclusions about the effectiveness of FG-cs because there is a lack of robust research allowing causal inferences, i.e., few (quasi) experimental or longitudinal studies have been conducted [1]. Studies tend to mainly focus on the implementation of the conferences and of the Family Group plans, and on participant satisfaction. In a study by Oosterkamp-Szwajcer et al. (2012), the majority of the participants indicated that the situation had improved after a FG-c, in 87% of the families the plans were carried out at least partially, and the family felt empowered [4]. However, given that no comparison group was included in this study, these positive results cannot be attributed to FG-c. In another Dutch study, Schuurman (2011) concluded that FG-c in 95% of the cases resulted in a plan, that participants were satisfied and that the situation had improved [5]. Schuurman and Mulder ( 2011) and Jagtenberg et al. (2011) argue that FG-cs save costs that otherwise would be spent on professional care [6,7]. Also in the research report provided by WESP (2008) all FG-cs resulted in a plan which, according to 44% of the members of the social network, was completely carried out [8]. None of these studies, however, used a comparison group, and thus results cannot with certainty be attributed to the use of FG-cs.

Research that did include a (not in all respects equivalent) comparison group [9], found that families that had participated in a FG-c reported less concerns about safety and well-being of the children and that social support from the network had increased. However, the changes in the Family Group-conferencing group were not larger than in the control group, and several methodological flaws of the study (e.g., short follow-up time, non-equivalence of the experimental and control group, high drop-out rates, lack of statistical power, and inadequate statistical analyses) did impose significant limitations on the causal inferences that could be drawn.

Notably, studies examining FG-cs show inconsistent results. Some researchers found positive effects of FG-c’s on short-term but no long-term effectiveness [10]. Where Burford et al. (2013) reported positive results of FG-c, Lorentzen (2009) and Sundell et al. (2004) did not find evidence for the effectiveness of FG-c’s [11-13]. Berzin et al. (2008) reported negative outcomes: families who had participated in FG-c had adverse outcomes for safety and stability of out of home placements and frequently refused necessary youth care [14]. A recent systematic review including only controlled studies, of which only six were available, showed that FG-c’s lead to an increase in child abuse, more and longer out of home placements and ultimately to an increase in use of professional care [15].

To conclude, although much research has been done to investigate FG-cs, there is still insufficient evidence for its effectiveness, in particular because of the use of weak study designs [16]. Research with a robust design does not confirm the positive findings reported in the uncontrolled studies [15]. Worldwide, only six studies including a control group have been conducted. It is therefore crucial to conduct a study that allows for conclusions on causality about the effects of FG-c’s.

Even less is known about potential moderators of the effectiveness of FG-cs: in which families, under what circumstances will family group conferences have the best results? Effectiveness studies try to find overall effects, whereas not all families are likely to benefit from one approach [17]. As Farrell, Meyer, Kung, and Sullivan (2001, p. 216) stated, “one cannot assume that an intervention program is equally effective with all participants” [18]. Therefore, analysis of overall program effects might suggest that programs did not work when in fact they did work for some subgroups [19]. Identifying moderators of the intervention effectiveness can be useful for identifying and engaging those who are most likely to benefit [20], and provides opportunities to adjust the programs to improve effectiveness for certain subgroups of clients.

In addition to potential moderating effects of family characteristics, program integrity may also moderate the effectiveness of FG-cs. An FG-c starts off with an initializing stage, than the actual conference takes place, and subsequently, the Family Group plan needs to be carried out. It is important to determine whether all stages belonging to a FG-c are carried out and to what extent the Family Group plan has been carried out as intended. This information can be used to determine which stages of the FG-c are critical for the achievement of potential positive results.

To increase the knowledge about the effectiveness of Family Group Conferences in youth care, we will perform a randomized controlled trial (RCT). In contrast to previous research that had several methodological shortcomings, this study will provide the opportunity to draw (scientifically robust) conclusions about the effectiveness of FG-c’s. In addition, it will become clear which characteristics of the approach, of the participating families and which characteristics of the family managers who should support the implementation of the Family Group plans are important to reach the best results. Finally, in times in which large amounts of public money are spent on ‘family autonomy and participation in child care’, operationalized in Family Group Conferences, it is important to base policy decisions concerning whether or not to continue those investments on clear numbers regarding the costs and benefits of Family Group Conferences. Therefore, the present effectiveness study will be accompanied by a cost-effectiveness analysis.

## Methods and design

The study aims to answer two main questions. The first question is whether Family Group conferences in youth care are effective as a decision making model in terms of increased child safety, involvement of the social network, perceived control over problems and a reduction in use of professional care. In order to determine the effectiveness of FG-cs, a RCT will be carried out including 300 families referred to Youth Care Agency Amsterdam (YCAA). Moreover, costs associated with FG-c will be identified in order to determine the cost-effectiveness of FG-cs.

The second research question concerns the moderators of the effectiveness of FG-c. Both participant and program characteristics will be examined as potential moderators. Program characteristics to be examined are program integrity and characteristics of FG coordinators and family managers (responsible for supporting the implementation of the FG plans developed by the broader social network of the family). It is crucial to examine program integrity (i.e., the extent to which different phases of a FG-c have been conducted according to the protocol). Without having established program integrity, there is the risk of concluding that FG-cs are not effective, whereas in fact the FG-c has not been fully implemented. Furthermore, it is possible that a successful FG-c has taken place, but the plan developed in the FG-c has never been carried out. It is therefore important to determine whether all stages of the FG-c have been implemented as planned.

Additionally, it is important to examine family characteristics as potential moderators of the effectiveness of FG-c. It is plausible that FG-cs are not equally effective for every participating family. For example, families with a small social network, or with limited possibilities to attend a conference or with other particular characteristics that hamper the organization or carrying out of a FG-c or the FG plans are less likely to attend a FG-c. Identifying those families who are less likely to fulfill an FG-c may result in savings.

### Design

A randomized Controlled trial will be conducted to examine whether FG-cs are effective in changing: (1) child’s safety (i.e., decrease in (risk for) abuse/neglect; less supervision orders or shorter supervision order; less or shorter out of home placement); (2) the involvement of the broader social network; (3) perceived control over the problems in the families, empowerment of parents; improvement of family functioning; (4) less use of professional care. A second aim is to study whether the effectiveness of FG-c is influenced by characteristics of the implementation of the methodology, characteristics of the family managers (such as education, or attitude towards FG-c), or by family characteristics (such as size of the social network, ethnicity or family type: single/married).

**Figure 1:**
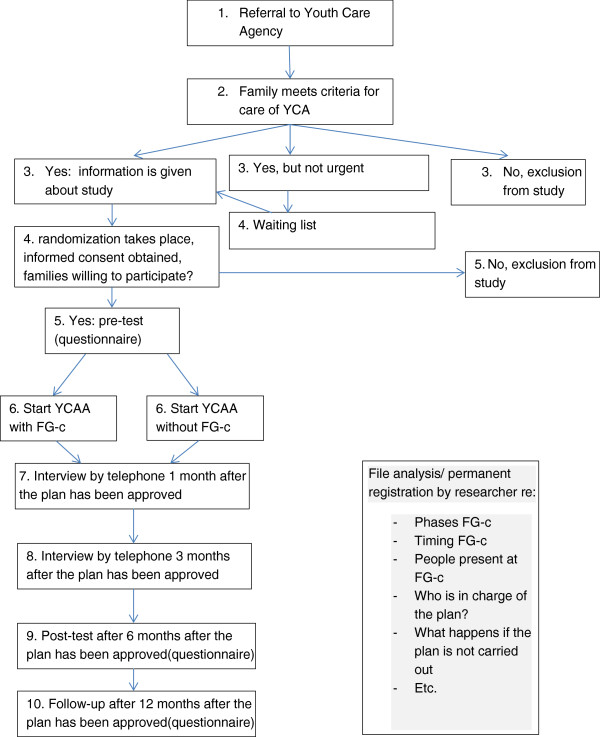
Randomization procedure.

Using computerized randomization two comparable groups will be formed. Until the inclusion of 300 families is completed (presumably within 6 months), every family starting care at Youth Care Agency Amsterdam (YCAA) will be randomly allocated to care with or without a Family Group conference. Because allocation to care with a FG-c will not always result in an actual Family Group conference and/or an approved Family Group plan, 200 families will be assigned to care with FG-c and 100 families to care without FG-c. Five multi-informant/source (parent, family manager; FG-coordinator; member of the family’s social network; child (if > 8 years of age), and file analysis) assessments will be carried out in both groups. Pre-tests will take place before FG-cs starts. Subsequently, in both groups post-tests will be carried out 1, 3, 6 and 12 months after the FG-c has taken place (see also Figure 1 for the flow chart).

In addition, for families in the FG-c condition, it will be assessed (1) whether a FG-c coordinator explained the concept of a FG-c; (2) whether an FG-c has taken place, (3) whether a plan has been formulated; and (4) whether the plan has been carried out (at the moments when posttests at 1, 3 and 6 months after the FG-c have been carried out). To diminish the burden that is imposed on families as much as possible, measurements at 1 and 3 months will be done as much as possible by telephone.

The design of this study is approved by the independent Ethical Committee of the Faculty of Social and Behavioral Sciences of The University of Amsterdam (approval number: 2013-POWL-3308).

### Study sample

We aim to include *N* = 300 families, who will be randomized in a 2 (FG-c): 1 (control group) ratio to ensure that there is enough power to also conduct the planned moderator analyses. These groups are large enough to detect effects of FG-c’s.

Given the goal of YCAA to carry out 800 FG-c’s each year, the inclusion of 300 families will be completed within six months. This sample size is sufficient to test the hypotheses with an alpha of .05 and a small to medium effect (*d* = .35). FG-c can be used for all families, so there are no exclusion criteria in this study.

### Recruitment

Participants are recruited at Youth Care Agency Amsterdam and Surroundings (YCAA). When a family is referred to YCAA, a first step is to establish whether the family indeed belongs to the target group of YCAA. If that is the case, a YCAA manager determines whether the need for help is urgent. If this is the case, the family is immediately assigned to a personal family manager and is informed about the study. In this stage, randomization takes place, active informed consent is received and both youth care and the study will start. If the situation is less urgent, families may be placed on the waiting list. For those families, informed consent, randomization and start of youth care will be postponed until the waiting list period has expired. Specifics on the flow of participants are graphically presented in Figure 1.

### Conditions

#### ***Family group- conference [in Dutch: Eigen Kracht-conferentie]***

A Family Group - conference (FG-c) is defined as an approach to care that allows citizens to keep responsibility over decisions on issues within the family, neighborhood, or group of people in which they operate (Oosterkamp Szwaycer et al., 2012). FG-c provides citizens the opportunity to make a plan, using their own capabilities and by using support from outside. This research will focus on FG-cs that are used in youth care as a decision-making model in which families together with others in their social network, such as extended family, neighbors, friends and others, make a plan to solve their problems. The responsibility for making the plan lies within the (extended) family and the social network. Professionals are involved only in order to determine whether the plans developed by the family group are sufficiently safe for the children and to provide information about professional help options.

The Program Bureau responsible for FG-cs (in Dutch: Eigen Kracht Centrale), a national organization that supports and performs FG-c’s, organizes the family group conferences. The FG-c Program Bureau organizes the recruitment and training of the FG coordinators and contracts FG coordinators for the duration of a conference. Family managers thus request a FG-c at the FG-c Program Bureau. The FG coordinator organizes the FG-c in close cooperation with the family and family manager. The FG coordinator is independently associated with the FG-c Program Bureau and is thus not employed by a professional youth care facility.

A FG-c consists of two phases: the first phase is preparation (activation phase): after referral (or self-referral) to a FG-c, the independent FG coordinator contacts stakeholders (family, important people for the family, professionals) and prepares the conference. The coordinator organizes a time and location in accordance with the family’s wishes and is responsible for all practical coordination. The second phase is the conference phase: professionals share information on the needs and care options during the actual conference. Additionally, professionals provide, if necessary, conditions for the FG plan. The meeting consists of the information phase, a private part and the presentation of the plan. During the private part, no professional youth care workers or FG coordinator is present. During the private part, the care plan is developed. During the presentation phase, the care plan is tested to ensure safety: the safety of the children in the family has to be guaranteed and approved upon by the family manager. If a plan has been developed, the FG-coordinator places the responsibility for implementation of the plan with the family and the broader social network.

The plan is the starting point for care and there is no other treatment plan next to it. The family manager monitors the implementation of the plan and ensures that the agreements are carried out as intended in contact with the responsible member of the social network. This procedure is similar to the usual case management conducted by YCAA, with the difference that with regard to an FG-c plan, the network is responsible for the implementation of the plan, whereas in usual care, this is the responsibility of the family manager. In addition, at each conference, there is a contact person (or more) from the social network of the family who contacts a family manager to organize a new FG-c when things do not proceed according to plan. Thus, both the social network and the ‘helpers’ (can be someone else than the family manager) have a role in the implementation of the plan, the process of cooperation and adaptation of the plan to the developments occurring after the conclusion of the plan.

### Control condition

In the control condition, the family will receive the usual care. This means that the family manager is the one who designs and organizes the care plan instead of the family. YCAA works according to Generic Family oriented method since 2011: child protection, juvenile probation and youth are no longer distinguished. Employees (former youth probation workers, youth care workers and child protection workers) have been retrained as family managers (FM), who are able to provide the needed care to a family (or by referring to other professional services). As in the experimental condition, in the control condition, family managers will also work with a care plan. The difference between the FG-c and the control condition lies in the manner in which the plan is established: In the control condition, this is done by the family manager, along with parents/family, they discuss problems in the family and formulate goals, strict agreements are made on what professional help will be started. Within the FG-c, the plan is made by the family and social network. In a Family Group plan, they explain how they see the solution to their problems and what steps they will undertake to solve the problems, including starting professional care. In the intervention and control conditions, the role of the family manager is actually the same: a family manager works in both conditions with the care plan that has been developed, however, the levels of control differ: in the FG-c condition, control over the plan is with the family, in the TAU condition, control is with the family manager.

### Instruments

An overview of the concepts, instruments and sources of information is presented in Table 1. At the 5 assessment points, the family manager, a member of the network, parent, the family group coordinator and the child (if older than 8 years) will be asked to answer questions, either online, or by telephone. The families and child will receive assistance when filling out the questionnaires.

**Table 1:** Concepts, instruments and informants at the different assessment points

	**Domain/concept**	**Instrument**	**Test**					**Source**					
			**T1**	**T2**	**T3**	**T4**	**T5**	**FM**	**MN**	**P**	**FGC**	**R**	**C**
Primary outcome													
	Safety	LIRIK	x		x	x	x	x	x				
		Safety line	x	x	x	x	x	x					
		CAPI	x		x		x	x	x	x			
		Succint		x		x	x	x	x	x			
Secondary outcomes													
	Commitment	PSQ	x	x	x	x	x	x		x			
		ISEL	x	x^1^	x^1^	x	x	x					
		Succint	x	x^1^	x^1^	x	x	x		x			
	Control/empowerment	Interview	x	x	x	x	x	x	x	x			
		Succint	x	x	x	x	x	x					
		FES	x	x^1^	x^1^	x	x	x					
	Family functioning	PSI	x^1^	x^1^	x^1^	x^1^	x^1^		x				
		Succint	x	x	x	x	x	x					
		FES	x	x^1^	x^1^	x	x	x					
	Family functioning	PSI	x^1^	x^1^	x^1^	x^1^	x^1^			x			
	Professional care	File research	x	x	x	x	x					x	
Cost- effectiveness	Costs	Cost questionnaire	x			x	x			x			
Moderators													
Characteristics implementation FG-c and family manager	Number of families	Registration form	x	x	x	x	x					x	
	Role FM	Registration form		x	x	x	x	x	x	x	x	x	
	Chacareristics FM	Registration form	x									x	
	Time to FG-c	File analysis	x	x								x	
	Number members network at FG-c	Registration form		x							x	x	
	Phases FG-c/plan carried out as intended	Registration form	x	x	x	x		x			x	x	
Characteristics families	Demograpgics	File research/questionnaire	x	x	x	x	x	x		x		x	
	Risk factors	Risk factors List	x					x		x		x	
	Problems child	BPC (if child > 8)	x			x	x						x

*Note*: T1 = pretest (questionnaire); T2 = post-test after 1 month (interview by telephone); T3 = post-test after 3 months (interview by telephone); T4 = post-test after 6 months (questionnaire); T5 = follow-up assessment after 12 months (questionnaire); FM = Family Manager; MN = member network; P = Parent; FG-c = Family Group-conference; FGC = Family Group Coordinator; R = researcher; C = Child; ^1^ = selection of the original instrument.

### Primary outcomes

The primary outcome is child safety. This outcome will be assessed by using various child safety indicators. First, to measure whether the safety in families has increased the Light Instrument to assess Risk of Child Abuse (Lirik) [21] will be used at all assessment points. This instrument is filled out by the Family manager. The Lirik is widely used in the Netherlands and is a standard procedure for the YCAA family managers.

Additionally, after each case management meeting, family managers fill out the ‘safety line’ of the family. Families are given a safety score between 1 and 10. This is a new measurement method, used by YCAA as standard practice. These multiple assessment points allow to detect changes in safety over time.

Furthermore, parents will be asked to fill out the Child Abuse Potential Inventory short version (CAPI) [22]. This is a widely used instrument to identify child abuse potential [23]. A Dutch version has been translated and validated by Grietens, De Haene, and Uyttebroek [24].

The “Success of Intervention Inventory”(Succint) will be used to evaluate the success of the intervention (care with or without FG-c). This questionnaire measures whether changes are visible in safety, perceived control and social support/cohesion. The Succint has been developed by PiResearch, especially to evaluate the success of FG-c [8]. This instrument will be filled out by a parent, family member, and family manager.

### Secondary outcomes

In order to assess the commitment of the *social network,* The Parenting Support Questionnaire (PSQ) [25] will be used to assess perceived support of the social network. Additionally, social support concerning well-being and housing will be assessed with the Interpersonal support evaluation questionnaire - short (ISEL) [26]. Additionally, the changes in social support will also be addressed with the Succint questionnaire [8].

The *perceived control* will be assessed with an interview concerning perceived control by the researcher. Parent, EK-coordinator, Family manager and a member of the network will be interviewed. Additionally, Perceived control will be assessed with the Succint [8]. Finally, empowerment of the parents will be assessed with the Family Empowerment Scale (FES) [27].

The *quality of family functioning* will be assessed with the Parenting Stress Index Short form [28]. This instrument assesses parental stress in child rearing situations. Additionally, family functioning in terms of problem definition and reason to search help is registered with the FG-checklist, an instrument standard used in the FG working procedure. Additionally, the Family Assessment Device (FAD) [29], consisting of 12 items, will be used to assess quality of family functioning. Moreover, file analysis will be used to identify family functioning issues.

Finally, Basic Needs Satisfaction in General Scale (BNSG-S) will be used to assess ’satisfaction of basic psychological needs’ [30]. This questionnaire consists of three subscales: autonomy, competence and social connectedness. This instrument is translated to Dutch [31] and validated for a mentally delayed population. Additionally, the Satisfaction with Life scale [32], consisting of 5 items, is used to assess general well-being of parents.

Finally, the use of professional care will be retrieved from files at each of the 5 assessment points. Trained coders will code the use of professional care.

### Cost-effectiveness

In addition to the primary and secondary outcomes, the costs of family group conferencing will be assessed with an adapted version of the questionnaire for Costs associated with Psychiatric Illness, developed by the Trimbos Institute (TiC-P) [33]. Items concerning social care and youth were added, as Jansen et al. (2013) did [34]. Both direct costs, those costs associated with the intervention (staff costs, overhead, contacts with others, travel costs) and direct cost associated with the youth care utilization (as indicated by parents) as well as indirect costs in terms of productivity loss will be assessed.

### Moderators

*Characteristics of the Family Group-conferences (FG-c)*, such as number of families per FG-coordinator, location, time to start of FG-c, number of members of the network present at the FG-c, will be registered on the registration form developed for this specific study.

Additionally, specific characteristics of the Family Group-conference, related to the *implementation of the FG-c*, will be registered on this form too, such as whether the separate phases of the FG-c have been followed as intended, duration of these phases, and whether the plan is carried out as intended and who was responsible for the carrying out of the plan, and whether this person took the assigned role.

Moreover, the *role of the family manager* in the carrying out of the FG-c will be monitored with the registration form: did the family manager bring the FG-c up within the intended period of time (2-6 weeks after start)? Was the family manager supportive of FG-c (and why (not))? Did the Family manager leave control with the family (and why (not))?

*Characteristics of the families* that may affect the effectiveness will be assessed. Demographics will be coded from the file research and information that is not present in the YCAA file will be asked with a questionnaire. Risk factors will be assessed with a risk list that consists of a combination of the Washington Prescreen Risk Assessment instrument [35], for the penal law cases and the Delta risk list specifically developed for civil law cases.

*Problems of the child* will be assessed with the Brief Problem Checklist [36], to be filled out by the parents (if the child is younger than 8 years of age) or the child (if the child is older than 8). This is a validated short questionnaire that assesses problems of the child.

### Statistical analyses

Missing data will be imputed so that all participants will be included in the analyses, and using LISREL 8.8, the multiple imputation will be carried out by the expectation maximization algorithm [37]. Both an intention to treat as well as a completer analysis will be performed.

In order to examine the effectiveness of FG-c overall effectiveness will be examined for all outcome measures by conducting an ANCOVA, with the outcome measures at post-test as dependent variables, treatment condition as factor and pre-intervention scores of the outcome variables as co-variates, as is recommended for RCTs using pre- and post-tests [38]. Effect sizes will be computed as Cohen’s d, based on adjusted means and standard errors.

For categorical moderator analyses, the same ANCOVA’s will be conducted, with the moderators as factor. Post-hoc analyses for moderator effects will be conducted by splitting the file according to the moderator and again conducting an ANCOVA and calculating effect sizes separately for each group. Regression analyses will be conducted for the continuous moderators.

## Discussion

The implementation of Family Group-conferencing for decision making in child welfare has gained popularity rapidly. In The Netherlands, a change in youth protection is proposed for 2015 according to which the law states that every family has the right to start child care with making their own plan. The philosophy of a society in which everyone can participate and has the right to make its own decisions is the background of this development. Question is, however, if every family is able to make its own plan, and if these plans are effective in terms of increased child safety, increased commitment of the social network, if families are able to gain control over their own help, if family functioning improves and use of professional care decreases. The present study addresses this by examining the effectiveness of FG-c by means of a randomized controlled trial. Worldwide, despite an overload of studies examining FG-c [16], there are only six studies that examined FG-c with a comparable comparison group [15]. It is thus of crucial importance to conduct a RCT of FG-c, including sufficient participants as the present study intends to achieve.

Moreover, few RCTs have been conducted of FG-c, even less so examine for whom, under what conditions FG-c is likely to be most effective, as the present study does by carefully examining implementation, method and participant characteristics.

The present study thus has several strenghts: several strengths: it will extend the knowledge on Family Group-conferencing as a decision making procedure in child care. By using a randomized controlled trial, confounding variables are excluded as much as possible. A sufficiently large sample size will be used to study effectiveness as well as moderators of the effectiveness.

Moreover, a multi-informant approach will be used by asking parents, family managers, FG coordinators, a member of the network and children (if older than 8) to report on the outcomes and by conducting file-analysis.

Additionally, a rich set of instruments will be used, so that if there is any effect on any of the outcomes, there is a large likelihood to identify this effect. Moreover, potential moderators in terms of program as well as participant characteristics will be examined, so that the present study will provide information on when and for whom FG-cs are most likely to be effective. Program integrity will be examined and fulfillment of each phase of FG-c will be registered. Finally, the cost-effectiveness of FG-c will be examined: valuable information for both politicians as well as families who invest time in a FG-c.

However, there are also several limitations, which may threaten the value of our study. First, there is a risk in having this many informants and assessment moments (5 time points). There is the risk that the families or other informants will drop out of the study. We will put a lot of effort in maintaining the families in the study. Thankfully, the research group has experience with motivating for and retaining participants in research [39], but this always needs full attention.

Additionally, TAU in the control group may cause smaller effect sizes when compared to no treatment in the control group. However, as the research question is whether FG-c adds something to the usual child care services, this is the most logical design.

## Conclusion

The present study aims to examine the (cost-) effectiveness of FG-c in The Netherlands. Given the wide popularity of FG-c and inconclusive research results this is of importance to all countries that implement FG-c with best intentions but without scientific proof of its effectiveness.

## Abbreviations

FG-c: Family Groups-conferencing/Family Group-conference; RCT: Randomized Controlled Trial; YCAA: Youth Care Agency Amsterdam and surroundings; FM: Family manager; CAPI: Child abuse potential inventory; ISEL: Interpersonal support evaluation questionnaire; FES: Family empowerment scale; FAD: Family assessment device; BNSG-S: Basic needs satisfaction in general scale; TiC-P: Trimbos Institute Questionnaire for Costs Associated with Psychiatric Illness; ANCOVA: Analysis of covariance; TAU: Treatment as usual.

## Competing interests

The authors declare that they have no competing interests.

## Authors’ contributions

JA, GJS, MD and HC obtained funding for the study and designed the trial. All authors contributed to the design of the study. SD and HC coordinate the recruitment of the participants and data collection during the study. JA wrote the manuscript, mainly based on the grant proposal written by JA, GJS, MD, and HC. All authors contributed to the writing of the manuscript. All authors read and approved the final manuscript.

## Pre-publication history

The pre-publication history for this paper can be accessed here:

http://www.biomedcentral.com/1471-2458/14/154/prepub
